# Ultra Structural Characterisation of Tetherin - a Protein Capable of Preventing Viral Release from the Plasma Membrane

**DOI:** 10.3390/v2040987

**Published:** 2010-04-12

**Authors:** Ravindra K. Gupta, Greg J. Towers

**Affiliations:** 1 Medical Research Council Centre for Medical Molecular Virology, University College London, London W1T4JF, UK; 2 Division of Infection and Immunity, University College London, 46 Cleveland Street, London W1T 4JF, UK

## Abstract

Tetherin is an antiviral restriction factor made by mammalian cells to protect them from viral infection. It prevents newly formed virus particles from leaving infected cells. Its antiviral mechanism appears to be remarkably uncomplicated. In 2 studies published in PLoS Pathogens electron microscopy is used to support the hypothesis that the tethers that link HIV-1 virions to tetherin expressing cells contain tetherin and are likely to contain tetherin alone. They also show that the HIV-1 encoded tetherin antagonist that is known to cause tetherin degradation, Vpu, serves to reduce the amount of tetherin in the particles thereby allowing their release.

Restriction factors are a class of interferon inducible proteins that are active against retroviruses as well as other unrelated viruses. They include TRIM5α, active against retroviruses [1] and recently described to be active against herpes viruses [2], and the APOBEC3 proteins APOBEC3G and APOBEC3F, which are active against retroviruses and hepatitis B virus [3,4], reviewed in [5]. In early 2008 a further restriction factor dubbed tetherin, previously referred to as BST2/CD317/HM1.24, was described [6,7]. Tetherin has antiviral activity against a broad range of unrelated viruses including retroviruses [6,7], filoviruses [8,9], arena viruses [9] and herpes viruses [10,11]. It has been demonstrated to tether nascent HIV-1 virions to the plasma membrane, preventing their release from the infected cell [6]. Subsequently, these virions are recruited back into the cell in endosomes and targeted to the lysozome [6,7,12,13]. Two recent papers in PLoS Pathogens [14,15] have attempted an ultrastructural characterization of tetherin and illustrated how it forms the tether between infected cell and nascent virus. Tetherin thus represents the altruistic activity of an infected cell to prevent viruses from leaving its membrane to infect new cells.

Tetherin is a type II integral membrane protein of between 28 and 36kDa, with a predicted trans-membrane and coiled coil regions as well as a predicted glycophospatidinylinositol (GPI) anchor site [16]. Thus both ends of tetherin are connected to the plasma membrane and both of these connections are required for restriction of HIV-1 [17]. Tetherin has also been shown to exist as a dimer and is glycosylated at two sites in its extracellular domain [16,18]. Dimerisation via cysteine cross links is also required for tethering function, as mutation of all three extracellular domain cysteine residues to alanine abrogates antiviral activity without altering tetherin’s surface expression levels [17,19]. Furthermore, N-linked glycosylation at position 92 but not 65 impacts on anti-HIV activity [19]. Some differences in tethering mechanisms between different viruses are suggested by the observation that N-linked glycosylation does not appear to impact on antiviral activity against Lassa or Marburg viruses [9].

In order to replicate in the presence of tetherin sensitive viruses are obliged to antagonize tetherin and remove it from the site of viral budding, generally the plasma membrane. Several groups have demonstrated that the HIV-1 viral protein U (Vpu) counteracts the antiviral activity of human tetherin [6,7], and that it does this in a species-specific manner. Vpu therefore acts against human tetherin but not tetherin from other species [20,21]. Since then a number of other virus encoded countermeasures have been described including the Nef protein from a variety of simian immunodeficiency viruses (SIVs) [22–24] the envelope glycoproteins from HIV-2 [25], SIV from Tantalus monkeys [21] and Ebola virus [26] as well as the RING CH ligase K5 of HHV-8 or Kaposi’s sarcoma associated herpes virus (KSHV) [10,11,27]. The envelope proteins from HIV-2 and SIVtan have also been shown to be species-specific antagonizing tetherins from the hosts in which they replicate but not distantly related tetherins [25,28]. Phylogenetic analyses have demonstrated clear evidence for positive selection during primate evolution in tetherin genes and point mutants at the positively selected residues impact on sensitivity to both Vpu and SIV envelope proteins. This suggests that similar Env/Vpu proteins might have provided selection pressure for tetherin evolutionary change during primate evolution [20,21,28].

Tetherin is expressed on the plasma membrane of a variety of cell lines, including HeLa, Molt4, H9, and Jurkat as well as primary T and B cells and macrophages [6,18,29,30]. It also localises to perinuclear compartments, notably the Trans Golgi Network (TGN) [16,31]. A model whereby tetherin continually cycles between the cell surface and the TGN with a proportion targeted for degradation whilst new molecules are translated, is most consistent with current data [32]. Human, murine and rat tetherins have been shown to localise to lipid microdomains or rafts at the plasma membrane [16,33,34] and both human and murine orthologues are internalized in a clathrin dependent pathway [32,34]. Rollason and colleagues also showed that this process involves a dual tyrosine motif in murine tetherin mediating binding to the AP1 and AP2 adaptor complex with subsequent internalisation towards the trans Golgi network [32].

Direct evidence that tetherin itself, tethers virions first came from Perez-Caballero and colleagues who showed through domain swapping and mutagenesis experiments that configuration rather than primary protein sequence was required for the tethering phenotype [17]. They were able to construct a protein with similar domain organization but no sequence homology to tetherin, which could also tether virions and was insensitive to HIV-1 Vpu. These investigators also presented evidence suggesting that tetherin is a parallel homodimer. They hypothesized that it incorporates into the viral membrane using either of its two membrane anchors, and that this configuration was sufficient for antiviral activity (Figure 1). It remains unclear whether the dimers of tetherin that span the plasma and viral membranes have a preference for which membrane anchor ends up in the cell membrane and which ends up in the virion although there is some evidence that the GPI anchor is favored in the cell membrane and the TM anchor in the virus [17] (Figure 1D). Deletion of either the TM domain or the GPI anchor abrogates antiviral activity suggesting that anti-parallel dimers with monomeric links to the membrane as shown in Figure 1 B do not exist or cannot effectively tether. However, several of the possible configurations shown in Figure 1 may co-exist and it will be difficult to prove that any particular configuration does not play a role in the antiviral tethers.

The two recent studies report the findings of immuno-electron microscopy studies confirming that tetherin is found in the physical bridge between nascent virions and the plasma membrane [14,15]. In agreement with Perez-Caballero and colleagues [17] they also find that tetherin co-sediments in sucrose gradients with viral particles produced by transfection of Vpu-deleted virus into cells stably expressing HA-tagged tetherin [14,15]. A number of investigators have seen large aggregations of virions apparently attached to one another [6,7] suggesting that tetherin in virion membranes can mediate virion-virion tethering. Using both transmission electron microscopy and cryosection electron microscopy Hammonds and colleagues investigate this using their own polyclonal rabbit anti-tetherin antisera and secondary goat anti-rabbit antibody conjugated to gold [14]. They show that the areas between adjacent virions in the chains stain for tetherin confirming that they are indeed linked by tetherin. Very low levels of background staining were found with these techniques with 91% of gold beads being found within 50nm of visible particles.

Interestingly, tetherin was not found uniformly on the plasma membrane. Instead, it was often found in foci some of which did not appear to be associated with any underlying structure. In some cases these foci appeared to be clathrin-coated pits, consistent with observations of AP2-mediated endocytosis of rodent tetherin associated with such structures [16]. The identity of the other concentrations is unclear but may represent preferred sites of budding that correspond to the cholesterol rich lipid microdomains or rafts that have previously been associated with tetherin [16,32]. A further intriguing finding was the observation of strong gold labeling in filamentous structures that connected the plasma membrane to virions at some distance. The filaments are too long to be explained by single tetherin dimers suggesting that they may contain membrane packed with tetherin molecules that contribute to the tethering mechanism. The authors argue that this is not an artefact of the sample preparation although the importance of this observation is yet to be fully elucidated. Together, these observations support the notion that tetherin is appropriately placed within the membrane to target viral budding.

Vpu appears to accelerate the net loss of active tetherin from the cell surface via βTRCP mediated ubiquitination and subsequent targeting to either the proteasome or lysosome, or both [35–37]. However, Fitzpatrick and colleagues used a virion capture assay using beads coated with anti-tetherin ectodomain antibody to show that Vpu does not entirely remove tetherin from virion envelopes [15]. This suggests that a small amount of tetherin can be tolerated on the virion surface and allow efficient budding. Most recently Habermann and colleagues have used quantitative immuno-EM and surprisingly found that tetherin is enriched relative to the plasma membrane in cell-free and cell associated virions and in viral buds in HeLa cells, independently of Vpu, supporting the notion that tetherin’s activity is dependent on its localisation within microdomains in the plasma membrane [38]. Haberman’s observations also show that some tetherin is found in free virions, despite the prescence of Vpu. Now that the ultra-structural details of tetherin’s direct action are better understood it will be interesting to see the results of experiments which follow the fate of tethered virions at such resolution. It will also be important to further elucidate the details of tetherin’s exact conformation in relation to plasma and viral membranes, as well as the homeostatic mechanisms regulating surface expression. We also look forward to *in vivo* studies that evaluate the functional importance of this intriguing protein.

## Figures and Tables

**Figure 1.**
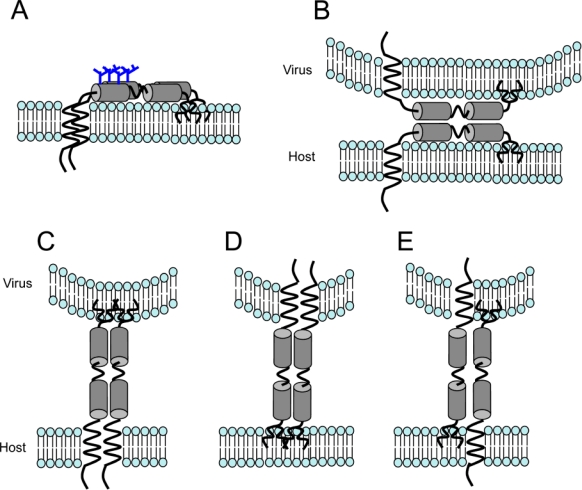
**Tetherin tethers newly formed virions to cells.** (A) Tetherin exists as a dimer in cell membranes. It tethers newly formed mature virions to infected cells preventing their release by forming tethers as the virus buds from the host cell membrane. The tethers may consist of parallel dimers (B-D) or anti-parallel dimers (E) although parallel dimers are likely to be favored [17]. It is unclear whether the parallel dimers preferably insert the GPI anchor (C) or the TM domain (D) into the virus membrane although both configurations may contribute to tethering. The fact that deleting either the GPI anchor or the TM domain prevents tethering suggests that either configurations B and E are not an important component of the tethering mechanism or that a single tethering domain is not enough to tether virions [17].
